# Emergence of CWD strains

**DOI:** 10.1007/s00441-022-03688-9

**Published:** 2022-10-06

**Authors:** Alicia Otero, Camilo Duque Velasquez, Debbie McKenzie, Judd Aiken

**Affiliations:** 1 Centro de Encefalopatias y Enfermedades Transmisibles Emergentes, University of Zaragoza, 50013 Zaragoza, Spain; 2 Department of Biological Science, University of Alberta, Edmonton, AB Canada; 3 Centre for Prions and Protein Folding Diseases, Brain and Aging Research Building, University of Alberta, Edmonton, AB T6G 2M8 Canada; 4 Department of Agriculture, Food & Nutritional Science, University of Alberta, Edmonton, AB Canada

**Keywords:** Chronic wasting disease, PrP structure, Prion strains

## Abstract

Chronic wasting disease (CWD) strains present a novel challenge to defining and mitigating this contagious prion disease of deer, elk, moose, and reindeer. Similar to strains of other prion diseases (bovine spongiform encephalopathy, sheep scrapie), CWD strains can affect biochemical and neuropathological properties of the infectious agent, and importantly interspecies transmission. To date, ten CWD strains have been characterized. The expanding range of CWD in North America and its presence in South Korea as well as Scandinavian countries will potentially result in millions of cervids infected with CWD; thus, novel strains will continue to emerge. In this review, we will summarize the characteristics of known CWD strains and describe the impact of prion protein gene polymorphisms on the generation of strains. We will also discuss the evidence that individual cervids can harbor more than one CWD strain, complicating strain analysis, and affecting selection and adaptation of strains in new hosts.

## Introduction

Fundamental to mitigating any disease epizootic is a thorough understanding of the biological properties of the infectious agent and its interaction with the host. Transmissible spongiform encephalopathies (TSEs) or prion diseases, in general, and chronic wasting disease (CWD) in cervids, in particular, represent significant challenges for disease management and eradication. These diseases, which include CWD, scrapie in sheep and goats, bovine spongiform encephalopathy (BSE) in cattle, and Creutzfeldt-Jakob disease (CJD) in humans, are caused by misfolded, infectious conformations (PrP^Sc^) of a host encoded (*PRNP* gene) cellular prion protein (PrP^C^; Prusiner 1982).

CWD is a contagious prion disease affecting captive and free-ranging elk, deer, moose, and reindeer. It was first identified in captive mule deer (*Odocoileus hemionus*) in Colorado in 1967 and was formally diagnosed as a transmissible spongiform encephalopathy in 1978 (Williams and Miller 2002; Williams 2005). Its host range has expanded into wild mule deer, white-tailed deer (*Odocoileus virginianus*), elk (*Cervus canadensis*), reindeer (*Rangifer tarandus*), and moose (*Alces alces*). The geographic range of CWD continues to increase with the disease reported in 4 Canadian provinces, 30 American states, South Korea, Norway, Finland, and Sweden.

The magnitude of the number of cervids currently infected with CWD and the number that will ultimately become infected is unprecedented. There are approximately 30 million white-tailed deer in the USA (https://www.wildlife.state.nh.us/wildlife/profiles/deer.html). Wyoming has an estimated deer population of 400,000 animals and a CWD prevalence, in chronically affected herds, of greater than 40% in deer and 15% in elk. The state of Wisconsin has an estimated white-tailed deer population of 1.5 million animals (https://dnr.wi.gov/wideermetrics/DeerStats.aspx?R=2). One Wisconsin county (Iowa county) has an estimated 221,000 deer with a CWD prevalence of ~30% in adult does and 40–50% of adult bucks. With the continuing expansion of geographic range and increasing disease prevalence, millions of cervids in North America are at risk of being CWD infected. In addition to having a significant impact on cervid health and populations, there will undoubtedly be additional, novel CWD strains emerging.

As with all prion diseases, CWD has a long pre-clinical phase (typically years) prior to the onset of clinical signs, making it difficult to readily distinguish infected from uninfected animals. The lack of ante-mortem tests further confounds identification of infected animals as well as development of mitigation strategies. Further confounding disease mitigation is the existence of prion disease variants, CWD strains. Prion strains are well documented in scrapie, BSE, and human prion diseases; however, comparatively few studies have examined CWD isolates for strain differences. In this review, we will summarize what is known about CWD strains as well as stress the importance of future studies characterizing the diversity of emerging CWD strains.

## PrP structure and prion strains

Recognized for decades, prion strains play a significant role in the biology of the transmissible spongiform encephalopathies. Strains are capable of stable propagation of their biological information upon passage. Prion strains can be distinguished based upon biochemical differences in the abnormal prion protein and length of incubation period and clinical symptoms as well as distribution and level of spongiform changes in the brains of infected animals. As a result, strain differences can influence important disease characteristics including interspecies transmission, ability to detect prion infections, and potential for the disease agent to survive inactivation and/or persist in the environment. The structure of the disease-associated form of the prion protein (PrP^Sc^) is undoubtedly the basis of prion strain differences and has been shown to differ between prion strains (reviewed in Block and Bartz 2022). Although not required for strain generation, the prion protein primary structure (i.e., amino acid sequence) can play an important role in strain generation and selection (Duque Velasquez et al. 2020; Hannaoui et al. 2017).

Prion strains were first described in goats (Stockman 1926) with scratching and nervous syndromes. The two syndromes were identified as distinct strains by Pattison and Millson (1961) who demonstrated that the distinct clinical signs observed with the isolates were retained upon serial passage. The pioneering work of Dickinson and Kimberlin demonstrated, through transmission experiments, strains of scrapie in laboratory rodents (reviewed by Bruce 1993). Strains have been identified in numerous prion diseases, including classical and atypical scrapie in sheep and goats (Pattison and Millson 1961; Benestad et al. 2003), classical and atypical forms of BSE in cattle (Wells et al. 1987; Casalone et al. 2004; Brown et al. 2006), sporadic and variant CJD in humans (Parchi et al. 2000; Telling et al. 1996; Bruce et al. 1997; Hill et al. 1997), and CWD in cervids (this review; Table 1).

**Table 1:** CWD strains

**Strain**	**PrP**^**CWD**^	**Neuropathological Features**	**Transmission to non-cervid hosts**^**a**^	**References**
Wisc-1	Unglycosylated band of 18–19 kDa detected with multiple antibodies High conformational stability and PK resistance	Widespread and symmetrical distribution of prion aggregates and gliosis	Syrian golden hamsters TgBeaver mice Bank voles	Duque Velasquez et al. (2015, 2020), Herbst et al. (2017, 2022), Hannaoui et al. (2017, 2021)
H95+	Unglycosylated band of 16–17 kDa detected with multiple antibodies High conformational stability. Low PK resistance	Localized deposition of prion aggregates affecting brain nuclei adjacent to the ventricular system—in tg60 mice expressing 96S-PrP^C^	Wild-type mice Syrian golden hamsters (poorly) TgBeaver mice	Duque Velasquez et al. (2015, 2020), Herbst et al. (2017, 2022)
CWD1	Non-distinguishable from Wisc-1	Symmetrical distribution of prion aggregates and gliosis. Intense spongiosis in hippocampus of Tg1536 mice		Angers et al. (2010)
CWD2	Non-distinguishable from Wisc-1 or CWD1	Asymmetrical distribution of prion aggregates and gliosis. Mild neuropathology in hippocampus of Tg1536 mice	TgBeaver mice	Angers et al. (2010), Herbst et al. (2017)
116G	Distinct from H95 when passaged in tg60 mice Low conformational stability and PK resistance	Intense spongiosis and PrP^CWD^ deposition in cortex, hippocampus and corpus callosum of tg1536 mice	Syrian golden hamsters TgBeaver mice Not transmissible to bank voles	Hannaoui et al. (2017, 2021), Herbst et al. (2017)
LL132	Unglycosylated band of ~18 kDa with 6H4 antibody High conformational stability	In elk: prominent intraneuronal and glial-associated PrP^CWD^ aggregates In tg12 mice: diffuse prion deposition in the neuropil. Mild neuropathology of the cerebellar granular layer		Moore et al. (2020)
Scandinavian Moose #1	Main C-terminal fragment of ~17 kDa; C-terminal fragment of ~13 kDa with SAF84 antibody; partial loss of N-terminal 12B2 epitope In bank voles: shorter incubation period than Scandinavian moose #2; 18 kDa band with low amount of 12–13 kDa band	In moose: PrP^CWD^ almost exclusively in intraneuronal aggregates. No staining with 12B2 and 9A2 antibodies. No lymphoid deposition In bank voles: intense neuropathology in hindbrain	Bank voles	Pirisinu et al. (2018), Nonno et al. (2020)
Scandinavian Moose #2	Main C-terminal fragment of ~17 kDa; C-terminal fragment of ~13 kDa with SAF84 antibody; partial loss of N-terminal 12B2 epitope In bank voles: longer incubation period than Scandinavian moose #1; 17-18 kDa band with high amount of 12-13 kDa band	In moose: PrP^CWD^ almost exclusively in intraneuronal aggregates. No staining with 12B2 and 9A2 antibodies. No lymphoid deposition In bank voles: mild to no neuropathology in hindbrain	Bank voles	Pirisinu et al. (2018), Nonno et al. (2020)
Scandinavian Red deer	PrPCWD is restricted to the CNS	In red deer: Immunostaining with N-terminal antibodies is weak with no intraneuronal deposition; similar to BSE	Ongoing	Vikoren et al. (2019), Tranulis et al. (2021)
Norwegian reindeer	PrPres glycosylation pattern indistinguishable from common North American strains Barely transmissible to bank voles—18kDa PrPres	In reindeer: Neuropathology indistinguishable from common North American CWD strains	Bank voles (poorly)	Benestad et al. (2016), Nonno et al. (2020)

^a^Only transmission to species different than cervids and in which the inoculated strain was characterized is included

For many decades, it was believed that TSE strains provided a compelling argument for the existence of a scrapie-specific nucleic acid, and that the protein-only (prion) hypothesis and the existence of prion strains were not compatible (Kimberlin 1982; Prusiner 1982). The first study linking TSE strain with PrP^Sc^ structure identified differences in morphology of scrapie-associated fibrils, sedimentation rate, and degree of proteinase K (PK) resistance when comparing the hamster-adapted 263K scrapie agent with two mouse-adapted scrapie agents, ME7 and 139A (Kascsak et al. 1985). Isolation, purification, and characterization of two strains of hamster-adapted transmissible mink encephalopathy further solidified the prion hypothesis by demonstrating that different prion protein structures dictated prion strains (Bessen and Marsh 1992). Two prion strains (hyper-HY and drowsy-DY) were shown to differ in protease sensitivity as well as the molecular weight of infectious (protease-resistant) prion protein. Cell-free conversion assays demonstrated that PrP^Sc^ purified from HY and DY brains imprinted their conformations during the conversion, resulting in the generation of strain-specific PK-resistant PrP^Sc^ (Bessen et al. 1995). The development of highly sensitive conformation-dependent immunoassay methods, capable of detecting differences in chaotrope-induced unfolding of PrP^C^ and PrP^Sc^, further strengthened the argument for protein structure underpinning prion strains (Safar et al. 1998; Safar et al. 2015).

Structural differences in the disease-associated PrP present in prion strains have been inferred from the biochemical analyses (described below), but recent advances in high-resolution cryo-EM are beginning to provide structural evidence for the link between prion structure and strains. A comparison of two different prion strains (263K from hamsters (Kraus et al. 2021) and RML from mice (Manka et al. 2022a, b) demonstrates that, although the two strains share a common PIRIBS structure, there are fundamental differences between the two (Telling 2022). A more direct comparison of two mouse strains (RML and ME7; Manka et al. 2022a, b) again identifies minor structural putative strain-specific differences in the PIRIBS structure.

### Methods for strain typing

Strain identification and characterization utilize a panel of assays to unequivocally designate a particular isolate as a strain. Traditionally, strain characterization requires animal passages, often several serial transmissions which could take years to complete. Strain properties, particularly incubation period, clinical signs, and neuropathology, should remain consistent and constant, upon serial passage, for a particular agent to be considered a bona fide strain. More recently, a number of ex vivo and in vitro assays have been developed which allow more rapid preliminary characterization of strains.

#### Animal bioassay

The most reliable method for the typing of CWD strains is bioassay in rodent panels. These rodent models can include “wild-type” rodents, i.e., non-transgenic mice, hamsters, and bank voles (reviewed in Otero et al. 2021a) as well as transgenic mice expressing different cervid PrP^C^ molecules (Meade-White et al. 2007; Angers et al. 2010, Bian et al. 2019; Duque Velasquez et al. 2015, 2020). Specific mouse lines have been instrumental in distinguishing different strains; one example is the tg60 line (expressing 96S allele of white-tailed deer PRNP) which does not favor efficient replication of the most common CWD strains in deer (Meade-White et al. 2007; Duque Velasquez et al. 2015; Otero et al. 2021b). Successful infection of tg60 mice provided definitive evidence of novel CWD strains (H95+ and 116AG; Duque Velasquez et al. 2015; Hannaoui et al. 2017, 2021). These bioassay studies are, however, both expensive and time-consuming due to the long incubation periods (months to years) and the requirement for serial passages in congenic hosts to establish the reproducibility of the disease phenotypes.

The transmission experiments provide critical information for strain typing including incubation period and clinical signs. Although these properties may be variable following first passage into a new host, they usually stabilize upon subsequent passage. As incubation period is dependent on titer of the starting inocula, neuropathological characterization of the infected brains provides strain-specific details (Fraser and Dickinson 1967). This method, referred to as lesion profile analysis, quantifies the extent of spongiosis across nine different regions of the brain.

PrP^Sc^ also accumulates in specific regions of the brain in a strain-specific manner. Two methods routinely used for analyzing PrP^Sc^ deposition are histoblot and scoring of PrPd in specific brain regions. Histoblot provides information as to gross differences in PrP^Sc^ deposition patterns (Taraboulos et al. 1992). PrP deposition profiling, described by Jeffrey and Gonzalez (2007), characterizes morphological patterns of deposition at the microscopic level (i.e., diffuse deposition versus aggregates) as a further means of differentiating scrapie strains.

#### Ex vivo assays

Two assays have been developed for ex vivo differentiation of strains. One is the standard scrapie cell assay (SSCA), which can be used as an alternative for mouse bioassay (van der Merwe et al. 2015). This cell-based technique was initially used for the study of mouse prion strains using the N2a cell line. Cells are incubated with prion-infected samples, cloned, and then the proportion of PrP^Sc^ positive cells quantified using automated counting equipment (Klohn et al. 2003). The development of the Elk21 cell line (expressing elk PrP^C^) allowed the evaluation of CWD prions (Bian et al. 2010). The SSCA provides more information than the cervid cell assay due to the number of different cell lines that are infectible by mouse prions (different cells preferentially amplify different mouse strains). The paucity of cell lines that efficiently replicate cervid prions somewhat limits the utility of the cervid cell assay.

Another ex vivo assay that can differentiate strains is the prion organotypic slice culture assay (POSCA). In this technique, brain slices are cultured and subsequently infected with prions. This analysis allows prion replication similar to that in intracerebral infections, but in an accelerated manner (Falsig and Aguzzi 2008). POSCA models for CWD have been developed using organotypic brain slices from transgenic mice expressing elk PrP (Tg12 line; Kondru et al. 2020).

#### In vitro methods

Strains can also often be distinguished based on biochemical differences of PrP^Sc^. Among the most important are PrP^Sc^ glycoforms, sensitivity to digestion by proteinase K (PK), and the western blot migration patterns of PK-resistant PrP (Bessen and Marsh 1992; Collinge et al. 1996; Parchi et al. 1996; Khalili-Shirazi et al. 2005). PrP^CWD^ (i.e., cervid PrP^Sc^) of some strains (Wisc-1, H95+, and 116AG) can be differentiated directly from brain homogenates from deer and transgenic mice by comparison of glycosylation profiles and SDS-PAGE migration patterns by western blot (Duque Velasquez et al. 2015; Hannaoui et al. 2017; Herbst et al. 2022); other strains (e.g., CWD1 and CWD2) do not exhibit overt biochemical differences (Angers et al. 2010).

CWD strains are encoded by different structures (conformers) of PrP^CWD^ which can be differentiated from each other based on their relative resistance to denaturation. Two assays are routinely used: conformation-dependent immunoassay (CDI) and conformation stability assay (CSA). CDI allows the quantification of PrP^C^ and PrP^Sc^ in samples from clinically affected tissue homogenates by exploiting the ability of anti-PrP antibodies to bind to exposed PrP^C^ epitopes, which are hidden in PrP^Sc^ and become exposed following denaturation in guanidine hydrochloride (GdnHCl). Denaturation of PrP^Sc^ before and after protease treatment allows for measurement of the concentration of both protease-sensitive and protease-resistant PrP^Sc^ (Safar et al. 1998, 2002). Sequential denaturation with increasing concentrations of GdnHCl provides the basis for conformational stability assay (CSA) allowing for comparison of the strain-specific multimeric PrP^Sc^ spectra (Haldiman et al. 2013; Duque Velasquez et al. 2020).

Atomic field flow field fractionation (AF4) fractionates prion particles by size. Analysis of the fractions provides information for specific prion preparations regarding hydrodynamic radius allowing one to correlate the relative size of prion particles with infectivity. Cortez et al. (2021) demonstrated that two hamster-adapted prion strains with distinct clinical presentation (hyper and drowsy; Bessen and Marsh 1992) differ in the size of the most infectious particle. Interestingly, both PrP^Sc^ aggregates become PK-resistant at the same size.

Two in vitro assays for amplifying prions have been developed. Both can amplify very small amounts of infectious prions (often sub-infectious doses), enhancing the detection of prions in tissues and in the environment. The prion misfolding cyclic amplification assay (PMCA) is a prion amplification technique in which PrP^C^ is converted into protease-resistant PrP through serial rounds of sonication and incubation (Saborio et al. 2001). PMCA can reproduce the intra- and interspecies transmission of CWD prions without any apparent alteration of CWD strain properties (Green et al. 2008). By using different PrP^C^ substrates in PMCA, it is possible to rapidly evaluate the transmission barrier of prions, as the relationship between host and substrate permits discrimination between prion strains. The real-time, quaking-induced conversion (RT-QuIC) assay measures the amplification characteristics (endpoint dilution, lag, and log phase) of prion strains comparing their seeding activity in different recombinant substrates (Schmitz et al. 2016). CWD strains such as 116AG show distinct RT-QuIC seeding activities compared to wild-type CWD prions, allowing strain typing (Hannaoui et al. 2017).

Although current in vitro analyses might suggest that a given sample (i.e., brain homogenate) contains a specific strain, verification currently requires passage in animals to establish strain-specific incubation periods, clinical signs, and neuropathology. Strain differences are confirmed upon serial passage in rodent panels (i.e., tg60 cervidized mice, gtE and gtK knock-in mouse lines, bank voles, tgCC mice expressing beaver PrP^C^). These studies are, however, time-consuming and expensive. Retrospective analysis of strains identified by animal passage will allow the identification of specific in vitro/biochemical signatures of strains. Strain characteristics can be faithfully recapitulated in PMCA (reviewed in Peden et al. 2021); a comprehensive comparison of established strains with novel isolates, in PMCA using a panel of substrates, would validate PMCA for strain identification and, thus, facilitate more rapid identification of strains.

## CWD strains

In early CWD studies, it was common for pooled isolates to be used as the inocula. This could result in loss or dilution of less common strains. Additionally, early publications often did not identify the source of the infectious brain homogenates (i.e., host species), the geographic location from which the samples were obtained, nor the PRNP genotype of the donor animal. More recent studies have emphasized characterization of individual isolates and several CWD strains have been identified; however, direct comparison of these agents is complicated by several factors. Strain characterization has used different rodent models (both “wild-type” and transgenic animals) which may express PrP^C^ at different levels. Taken together, strain data from different research groups is often not directly comparable. Strain characterization, to date, however, has identified at least 10 distinct CWD strains (Table 1).

### Early evidence of CWD strains

Early evidence for CWD strains utilized a cell-free conversion assay to determine susceptibility of various rodent species to CWD (Raymond et al. 2000). Based on the cell-free conversion data, these isolates were then used to infect five species of hamsters, transgenic mice expressing hamster prion protein, and two non-transgenic strains of mice. There was variability in the transmission of CWD isolates from a variety of cervids to this rodent panel. When brains from clinically affected hamsters were serially passaged in Syrian Golden hamsters, two strains appeared; these strains varied in stable incubation periods (85–89 days vs 408–544 days) and had distinct neuropathology (Raymond et al. 2007).

Ferrets (*Mustela putorius furo*) are also a valuable animal model for the study of CWD (Bartz et al. 1998; Sigurdson et al. 2008). Transmission of two sources of CWD showed distinct and reproducible pathological phenotypes after adaptation to ferrets. These two ferret strains, named CWD-UWI and CWD-CSU, had distinctly different sensitivities to proteinase K digestion and differential distribution of PrP^CWD^ in the brain and lymphoid organs (Perrott et al. 2012).

Bioassays of CWD isolates from captive and wild cervids from different North American locations into transgenic mice overexpressing deer PrP (Tg(CerPrP)1536^+/−^ mice) provided evidence for two CWD prion strains, denoted CWD1 and CWD2 (Angers et al. 2010). Although primary transmissions of prions from elk produced either CWD1 or CWD2 phenotypes, the inoculation of deer CWD in the transgenic mice generated mixed survival times and neuropathological features. The CWD1 strain has relatively short incubation times (~200dpi), intense vacuolation of the hippocampus and widespread, symmetrical PrP^CWD^ deposition, and astrocytic gliosis in Tg(CerPrP)1536^+/−^ mice. CWD2 is, however, characterized by longer incubation periods (~300dpi), low vacuolation scores in the hippocampus, and asymmetrical distribution of PrP^CWD^, affecting only the inoculated hemisphere. Despite these differences, PrP^CWD^ from both strains have indistinguishable glycosylation patterns, and very similar guanidine hydrochloride denaturation profiles (Angers et al. 2010). Taken together, these results suggest that deer brains contain CWD1/CWD2 mixtures, whereas elk are usually infected with either one of the strains. Serial transmission of elk prions, however, favored strain mixtures (i.e., mixed profiles were observed in the transgenic mice upon second passage).

### PRNP polymorphisms affect strain generation

**Fig. 1:**
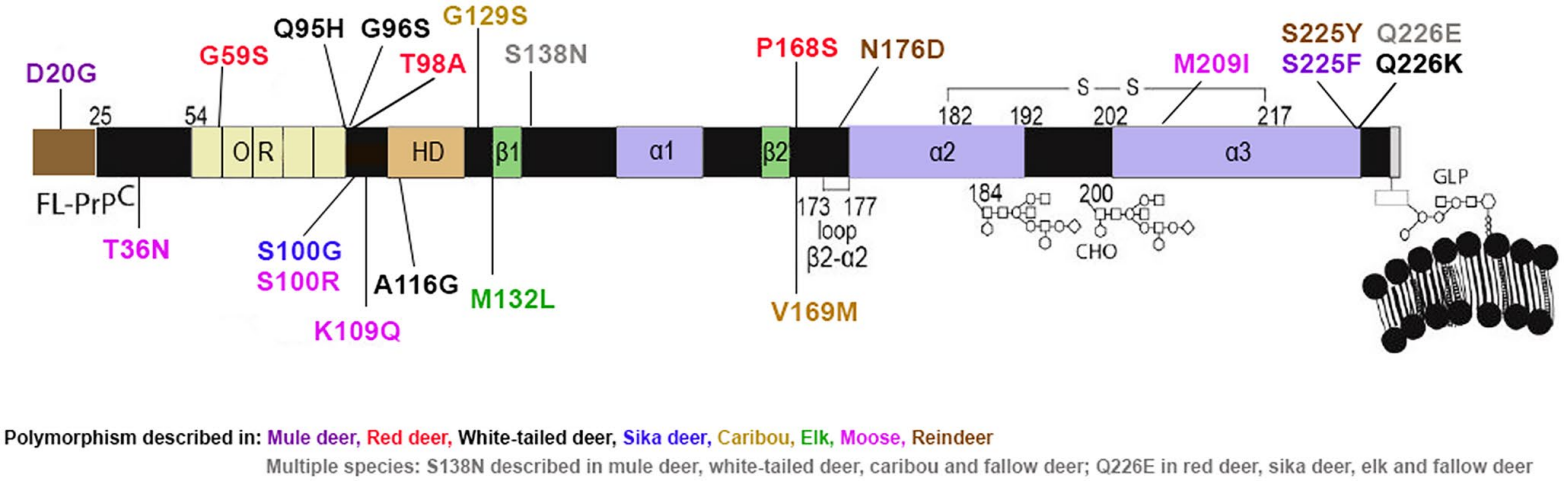
Cervid PrP^C^ representation showing the main polymorphisms in cervid species. FL-PrP^C^ : Full length PrP^C^; OR: Octarepeat region, HD: Hydrophobic domain; β1 and 2: β sheets 1 and 2; α1, 2 and 3: α helices 1, 2 and 3; GLP: Glycolipid

Although the *PRNP* gene is highly conserved in the Cervidae family, several allele variants have been identified encoding PrP^C^ molecules with different amino acid sequences (Fig. 1). In white-tailed deer populations, the most common PRNP allele (wt) codes for glutamine (Q), glycine (G), alanine (A), and glutamine (Q) at amino acids 95, 96, 116 and 226, respectively. Various PRNP alleles coding for amino acid substitutions, however, have been identified, such as Q95H, G96S, A116G, and Q226K (Raymond et al. 2000; Heaton et al. 2003; Johnson et al. 2003; O'Rourke et al. 2004).

Differences between primary amino acid sequence of PrP^CWD^ from the donor and PrP^C^ of the recipient host are the main determinants of the transmission barrier and, consequently, of strain generation (Scott et al. 1989; Prusiner et al. 1990; Bessen and Marsh 1992; Bartz et al. 1994, 2000). PrP^C^ polymorphisms not only dictate susceptibility to CWD but also modulate strain selection, resulting in the emergence and adaptation of new CWD strains (Duque Velasquez et al. 2015, Hannaoui et al. 2017). Nonhomologous PrP^C^ substrates modulate the selection and replication fidelity of the invading strain conformer (conformation expansion) affecting the biological stability and resulting in emergence of new strains through competitive selection of new conformers (Duque Velasquez et al. 2020). As we explore in the section below, certain PRNP polymorphisms of cervids including white-tailed deer 95H and 116G, elk 132L, and the amino acid difference at codon 226 between deer and elk PrP^C^ sequences are directly implicated in the propagation of distinct CWD strains (Duque Velasquez et al. 2015; Hannaoui et al. 2017; Bian et al. 2019).

### Wisc-1/H95+

Experimental infection of white-tailed deer expressing Q95/G96 (wt/wt), S96/wt, H95/wt or H95/S96 PrP^C^ molecules confirmed the impact of PRNP polymorphisms on the incubation period (Johnson et al. 2011). Homozygous wt/wt deer had a relatively short incubation period, succumbing to CWD with an average survival of 23 months post-infection (mpi). Heterozygous deer S96/wt, H95/wt, and H95/S96 deer had longer incubation periods suggesting partial resistance to disease. Interestingly, H95-PrPC impacted disease progression the most as heterozygous S96/wt deer had an average survival of 32 mpi, and deer expressing H95-PrPC survived longer than 50 mpi (Johnson et al. 2011) with altered peripheral distribution of prions in these animals (Otero et al. 2019).

Passage of these deer isolates in tg33 (wt) and tg60 (S96) transgenic mouse lines provided further insights into these agents. Neuropathological profiles and PrPres type in tg33 mice were similar irrespective of the CWD lineage they received, and their PrPres conformational stability highly resembled wt/wt deer CWD prions, demonstrating adaptation of a common strain to all the lineages (denoted as Wisc-1). Differences in incubation period after first passage of H95/S96 into tg33 mice were attributed to a loss of Wisc-1 specific titer following replication with H95 or S96-PrP^C^ (Duque Velasquez et al. 2015, 2020). The Wisc-1 conformer was maintained upon conversion of H95 and S96-PrP^C^ and could be reselected when transmitted to a host expressing wt-PrP^C^.

Host strain selection in transgenic mice expressing S96-PrP^C^ resulted in selection and adaptation of a distinct strain (H95+). Only the H95/wt and H95/S96 CWD isolates resulted in prion disease when inoculated into tg60 mice, with incubation periods that shorten upon serial passage (Duque Velasquez et al. 2015, 2020). The conformational stability of the H95+ PrP^CWD^, propagated in tg60 mice, is indistinguishable from the core of the H95/S96 deer PrP^CWD^ demonstrating the emergence of a new strain. When H95+ strain, passaged in tg60 mice, was used to infect tg33 mice (expressing wt-PrP^C^), similar neuropathological and PrP^CWD^ properties were observed, demonstrating the ability of H95+ to stably replicate with wt-PrP^C^ (Duque Velasquez et al. 2015). The opposite was also possible; brain homogenates from tg33 mice infected with H95/S96 CWD prions resulted in selection of H95+ when inoculated into tg60 mice (Duque Velasquez et al. 2015; 2020). These tg33 donors, despite being classified, based on histopathological profiles and by western blot, as Wisc-1, contained a mixture of PrP^CWD^ populations with distinct conformational stability compared to tg33 mice exposed to wt/wt prions, also incapable of producing disease in tg60 mice (Duque Velasquez et al. 2020).

### Gene-targeted mice expressing either E226 or Q226 PrP differentiate CWD strains

The primary sequence of elk and deer PrP differs by one amino acid at codon 226 (Robinson et al. 2012). When gene-targeted mice, GtE226^+/+^ and GtQ226^+/+^ mice (expressing elk and deer PrP^C^, respectively), were challenged with isolates from CWD-affected North American deer and elk (Bian et al. 2019), survival periods were shorter in GtE226^+/+^ than in GtQ226^+/+^ regardless of the inocula used, correlating with the observations in other mouse models for CWD (Browning and Mason 2004; Kong et al. 2005; LaFauci et al. 2006; Angers et al. 2009). Two main patterns of PrP^CWD^ deposition were observed. One pattern, observed in all GtQ226 mice and in GtE226 mice challenged with deer prions, was characterized by asymmetrical and disorganized prion deposits, ipsilateral to the inoculation site (Bian et al. 2019), like that observed for the CWD2 strain (Angers et al. 2010). The second PrP^d^ pattern was similar to the CWD1 strain with diffuse and bilaterally symmetrical prion deposits, it was only observed in GtE226^+/+^ mice challenged with elk prions. The results suggest that the strain properties of CWD prions generated in elk are distinct from those of prions producing disease in deer and that residue 226 selects and propagates distinct strains from mixtures of PrP^CWD^ conformers (Bian et al. 2019).

### Deer heterozygous for A116G PRNP replicate a novel CWD strain

Hannaoui et al. (2017) characterized the effect of the A116G PrP^C^ polymorphism of WTD comparing Wisc-1 CWD (homozygous A116; wild-type PrP^C^) with prions obtained from a A116G heterozygous, hunter-harvested wild deer. This amino acid substitution is in the hydrophobic core region of PrP^C^. In silico analysis suggests that the structure of G116 PrP^C^ is less stable and more flexible than A116 PrP^C^ (Hannaoui et al. 2017). Compared to Wisc-1, 116AG prions show lower conformational stability in GdnHCl, lower resistance to proteases, and decreased RT-QuIC seeding activity characterized by extended lag and log phases. In addition, 116AG prions were less efficient propagating in primary neuronal cultures and produced long incubation periods upon first passage into tg1536+/+ mice suggesting new conformational variants were amplified during prion replication in the 116AG deer. After the second passage, however, survival periods of 116AG inoculated mice were significantly reduced, and most mice had shorter disease progression times compared to mice inoculated with Wisc-1 prions. Differences between 116AG and Wisc-1 prions were maintained after passage in transgenic mice, demonstrating that 116AG deer contains a distinct CWD strain (Hannaoui et al. 2017). It remains to be determined if the 116AG strain was generated by conformational diversification under the selective pressure of the G116-PrP^C^ or selected from a pre-existent mixture acquired by the 116AG deer in the wild. Isolates from wild cervids naturally infected with CWD and sheep and goats with scrapie have been shown to contain prion strain mixtures (Angers et al. 2010; Barrio et al. 2020; Nonno et al. 2020).

### Elk strains

PRNP in elk is variable at amino acid 132, methionine and leucine. Experimental CWD transmission in elk expressing the three different 132 PRNP genotypes resulted in differential CWD progression with MM132 elk succumbing first, followed by ML132 elk and finally LL132 elk. LL132 elk have differences in the distribution of CWD lesions in their brains and the PrP^CWD^ showed a distinct protease-resistant PrP^CWD^ pattern (O'Rourke et al. 2007) as well as distinct fibril stability, abundance in the brain, and RT-QuIC seeding activity compared to MM132 and ML132 elk prions (Moore et al. 2018). Strain typing of these isolates in transgenic mice expressing MM132 elk PrP^C^ (Tg12 mice) results in prolonged incubation periods for LL132 isolates, and the PrP^CWD^ generated in the mice showed reduced accumulation in the brain and lower molecular weight than the PrP^CWD^ detected in mice inoculated with MM132 or ML132 brain homogenates. No overt differences were observed in the disease phenotype between mice inoculated with MM132 or ML132 CWD. The phenotypic features observed in experimentally infected elk were maintained after two passages of LL132, MM132, and ML132 isolates into mice expressing MM132 PrP^C^. Thus, CWD prions generated in LL132 elk represent a novel CWD strain (Moore et al. 2020).

Whether the prion strain generated in LL132 elk is also present in elk expressing ML132 PrP^C^ remains to be elucidated. Although ML132 and MM132 elk prions had indistinguishable neuropathological characteristics and fibril stability, ML132 elk had intermediate incubation periods, less PrP^CWD^ in the brain, and different seeding activity of the generated prions compared with MM132 and LL132 elk (Moore et al. 2018). Differences in incubation periods and amount of prions in the brain between ML132 and MM132 elk may be explained by the lower expression of M132 PrP^C^ in the heterozygous animals compared to the homozygous animals, and that M132 is more permissive to prion propagation than L132 PrP^C^ (Green et al. 2008; Moore et al. 2018). Second passage of ML132 prions in transgenic mice, however, resulted in two different groups with differences in incubation periods and prion fibril stability suggesting that the ML132 elk isolate might contain a mixture of prion conformations (Moore et al. 2020).

### CWD in reindeer

*Rangifer tarandus* sp. is present in Northern Europe (European reindeer) and North America (Barren ground caribou, woodland caribou, Porcupine caribou, and Peary caribou). PrP^C^ polymorphisms have been identified in European reindeer: G129S, S138N, V169M, N176D, and S225Y (Mitchell et al. 2012; Wik et al. 2012). Sequencing of the reindeer PRNP alleles indicated that SS225-PrP^C^ and/or a PRNP octapeptide deletion were overrepresented among the CWD-positive animals (Guere et al. 2020). Genetic analyses of three caribou herds from Alaska revealed four PRNP alleles coding for different PrP^C^ amino acid polymorphisms: V2M, G129S, S138N, and V169M (Happ et al. 2007); Canadian caribou had additional polymorphisms at Y153F and P242L (Arifin et al. 2020). Experimental oral CWD infections of reindeer showed that variation at position S138N apparently influences CWD susceptibility. Mitchell and colleagues suggested that the SS138 genotype was associated with susceptibility to CWD and the SN138 genotype with resistance (Mitchell et al. 2012). It was later shown that SN138 reindeer can develop disease, but PrP^CWD^ accumulation in their lymphoid tissues was significantly lower than in reindeer expressing SS138 or NN138 PrP^C^ (Moore et al. 2018).

### CWD in moose

North American and European moose replicate different strains of CWD. Polymorphisms at positions 36 encoding for threonine (T) or asparagine (N) and 209 encoding for methionine (M) or isoleucine (I) have been described in North American moose (Huson and Happ 2006; Wik et al. 2012). In addition, the unique amino acid variant K109Q (K=lysine) was detected in European moose (Wik et al. 2012). CWD cases in moose are less abundant than in other cervids. All positive cases in free-ranging moose have been detected in animals of the wild-type genotype (KK109, MM209) (Baeten et al. 2007; Pirisinu et al. 2018). Currently, the number of CWD-positive moose is too low to determine if PrP- amino acid variation affects susceptibility to CWD. During experimental transmission of CWD to MM209 and MI209 moose, abnormal PrP aggregates occurred in brain and lymph tissues at 15.5 and 18.9 mpi, respectively; however, it is not possible to draw conclusions about the effect of PrP^C^ primary sequence as these animals died without showing clinical signs of CWD (Kreeger et al. 2006). The relative abundance of these two *PRNP* alleles and their effect on CWD susceptibility and progression remains to be determined.

In 2018, Pirisinu and colleagues reported three positive cases of CWD in old female moose (13, 14 and 13 years old) from Norway. Interestingly, PrP^CWD^ in these animals differed from moose CWD from Canada, reindeer CWD from Scandinavia, and other prion diseases. CWD in Norwegian moose is characterized by intraneuronal prion aggregates, absence of lymphoid tissue involvement, and a low molecular weight PrP^CWD^ with partial loss of the epitope recognized by the 12B2 anti-prion antibody (amino acids 93–97, cervid numbering). These features clearly differ from Norwegian reindeer CWD (Benestad et al. 2016). CWD was later detected in five additional moose in Norway, three moose from Sweden (16, 16, and 9 years old, respectively), and two, greater than 10-year-old, moose from Finland (Agren et al. 2021; Tranulis et al. 2021). Preliminary characterization of the Swedish and Finnish moose shows a similar presentation to Norwegian moose CWD (Gavier‐Widen 2019; Korpenfelt 2019; Koutsoumanis et al. 2019; Tranulis et al. 2021). North American moose PrP^CWD^ is indistinguishable, by western blot, from PrP^CWD^ found in other CWD-infected North American cervids (Kreeger et al. 2006; Baeten et al. 2007) and there are no differences between the primary sequences of CWD-positive reindeer and moose PrP. This suggests that the unique characteristics of Scandinavian moose CWD are not due to the species nor amino acid variations of PrP and that it represents a novel CWD strain (Pirisinu et al. 2018). The epidemiological characteristics (i.e., isolated cases found in aged animals) and the phenotypic features (i.e., CWD prions are restricted to the CNS) of CWD in European moose suggest it could be a spontaneous prion disease, has been hypothesized for atypical/Nor98 scrapie (Benestad et al. 2008; Fediaevsky et al. 2010).

The discovery of CWD in European cervids in 2016 led to multiple questions about the possible origin of the disease in the continent (Benestad et al. 2016). A strain typing study in which different CWD isolates were transmitted to bank voles was performed to determine whether European and North American CWD cases were associated. Surprisingly, adaptation of the CWD isolates to bank voles showed that CWD prions from Europe are distinct from those found in North America and that at least four different CWD strains can be differentiated: one, common to all Canadian CWD cases, one from Norwegian reindeer CWD (R-NO1), and two distinct strains in Norwegian moose (M-NO1 and M-NO2) (Nonno et al. 2020). Strain typing of European CWD isolates is ongoing, and it is important to assess the zoonotic ability of these CWD prions.

## Interspecies transmission

One of the more important characteristics that can define a strain is interspecies transmission. Success of an interspecies transmission is dependent on the interactions of the PrP^Sc^ with the PrP^C^ of the new host; these parameters are not, however, readily predictable. The species barrier effect and interspecies transmission have not been thoroughly investigated at the level of CWD strains. Although CWD has been successfully transmitted to several other species (Table 1 and reviewed in Otero et al. 2021a), individual strains have not been extensively analyzed.

Evidence for interspecies transmission resulting in emergent CWD strains was observed upon adaptation of CWD into ferrets (*Mustela putorius furo*). Mule deer CWD passaged through ferrets causes clinical disease in Syrian hamsters (*Mesocricetus auratus*), while the initial mule deer CWD inocula did not (Bartz et al. 1998). Passage of CWD through transgenic mice expressing hamster *PRNP* followed by passage in hamsters resulted in the isolation of two distinct phenotypes based on incubation periods and clinical signs (Crowell et al. 2015). These two observed phenotypes, termed cheeky (CKY) for the development of incessant hiccips and wasted (WST) for the dramatic weight loss upon onset of clinical disease, were subsequently determined to be two distinct strains, demonstrating that cross-species CWD transmission can result in new strains (Crowell et al. 2015).

An expanded host range was also observed with the H95+ strain. When C57Bl/6 mice and Syrian golden hamsters were intracerebrally infected with the Wisc-1 and H95+ strains, differential transmission patterns were observed. We, and others, have demonstrated that C57Bl/6 mice are not susceptible to infection with common CWD strains (Herbst et al. 2017). H95+, however, resulted in clinical infection of this mouse line. Conversely, hamsters were susceptible to the Wisc-1 strain but had limited susceptibility to H95+.

In another study testing interspecies transmission of CWD, challenge of a transgenic mouse line expressing beaver *PRNP* with a range of CWD strains showed no species barrier. There were, however, strain-specific differences in incubation periods and in the biochemical properties of the CWD prions generated in the transgenic beaver mice (Herbst et al. 2022).

### Strain adaptation, selection, and evolution

The concept of strain adaptation, selection, and evolution was first described in mice infected with sheep scrapie isolates, prior to the discovery of the prion protein (reviewed by Dickinson and Meikle 1970; Bruce and Dickinson 1979; Kimberlin 1982). Several different types of biological stability were described, based on the “visible” properties of the disease (incubation periods, neuropathology, clinical signs). In general, upon serial passage of a specific isolate in a new host, the incubation period, clinical signs, and neuropathology stabilize, this is considered adaptation to a new host. Passage of some isolates into a new host resulted in different strain properties (i.e., different incubation periods, neuropathology, and clinical signs following adaptation to the new host) suggesting selection or mutation of the original strain.

With the discovery of the prion protein, these observations can be linked to changes in prion conformation/structure. Upon primary passage into a new host, the PrP^Sc^:PrP^C^ interaction varies depending on the homology between the two. With serial passage, the selection for the most favorable conformation for conversion is selected, with a resulting decrease in incubation period, stabilization of neuropathology (i.e., deposition of PrPd and lesion profiles), and clinical signs. This stabilization of strain properties can also result in changes in the biochemical properties of PrP^Sc^ (guanidine stability, sensitivity to proteinase K, migration patterns on SDS-Page).

Field isolates of scrapie can contain more than one strain, i.e., more than one conformation that can be converted to PrP^Sc^. These conformers can then compete for PrP^C^, resulting in variable incubation periods. Subsequent passages, particularly with low titers of PrP^Sc^, will lead for the selection of one strain or the other. This was nicely demonstrated by Bartz et al. (2000) with transmission of mink transmissible encephalopathy into hamsters. On the initial passage, the incubation period was long and western blot analysis showed two different sized PrP^Sc^ migration patterns. Low titer passage then resulted in the adaptation and selection of two different strains (hyperactive, HY; and drowsy, DY) with significantly different incubation periods and two different PrP^Sc^ structures (Bartz et al. 2000; Caughey﻿ et al. 1998; Safar et al. 1998; Cortez et al. 2021).

Several models have been proposed to explain the process of strain selection. Prion strains have been proposed to exist as quasispecies or clouds of conformational variants that “mutate” and are selected based on compatibility with the host PrP^C^ (Li et al. 2010; Collinge 2010). Different host PrP^C^ molecules would be compatible with only a subset of the PrP^Sc^ conformers, resulting in the selection and adaptation of the infectious agent. Another model, deformed templating mechanism suggests that inaccurate matching of the host PrP^C^ and incoming PrP^Sc^ results in the generation of novel conformers (Baskakov 2014). Despite all the evidence for the link between PrP^Sc^ conformation and strain, there is, to date, no data directly measuring the conformational diversity, frequency, and mutation rate of conformers in an infectious prion preparation.

As described for scrapie strains, CWD strains undergo adaptation, selection, and evolution in a strain-specific manner. Transmission of CWD prions into white-tailed deer expressing amino acid polymorphisms (H95 and/or S96) resulted in the prion conformation expansion (and the formation of strain mixtures in deer heterologous for H95 (Duque Velasquez et al. 2015, 2020). Passage of these CWD lineages into the tg60 mice (expressing S96-PrPC) resulted in the selection and adaption of the H95+ strain accompanied by the reduction of conformational heterogeneity, buildup of higher infections titers, and stabilization of phenotypic characteristics. By comparison, passage in tg33 mice (expressing G96-PrPC) resulted in selection of Wisc-1. Transmission of CWD lineages from deer that do not express H95+ did not result in clinical disease but amplified non-adaptive S96 conformers. None of these lineages (Wisc-1, etc.), upon passage in tg60 mice, contained H95+ conformers based on both serial PMCA in S96-PrPC substrate and bioassay in tg60 mice (Otero et al. 2021b; Duque Velasquez et al. 2020). Together, these experiments suggest that the H95+ conformers efficiently convert S96-PrP^C^, while the Wisc-1 conformer loses the ability to convert S96-PrP^C^ with passage.

Another excellent system for characterization of strain evolution is the gene-targeted mice generated by the Telling lab. As described above, different strains/sources of CWD agent are more readily transmitted in either the GtE or the GtQ mice. Of interest is the serial passage of moose strains from Norway in these mice (Bian et al. 2019, 2021). The Scandinavian moose strains have very different properties than North American moose strains, with no apparent involvement of the lymph system. Serial passage of these agents in GtQ mice, however, results in the selection/adaptation of prion conformers such that the isolates more resemble North American CWD lineages. These data suggest that the North American CWD strains (i.e., Wisc-1) have also evolved, through numerous passages, to a few dominant strains.

Understanding strain selection and adaptation in cervids is complicated by the presence of more than one strain/conformer in free-ranging CWD-positive deer. In many areas where CWD is endemic, an individual animal may be exposed to CWD from either different cervid species or the same species expressing different PrP^C^ molecules. The passage history is unknown for naturally infected animals so it is difficult to assess the strain composition in a given animal. Characterization of isolates from CWD-infected cervids suggests that many are comprised of strain mixtures. Passage into a specific host results in the selection of the most favored conformation. Subsequent serial passages in the same host result in host adaptation (seen as a reduction in incubation periods). In our experience, these mixtures complicate strain characterization as passage through a panel of rodent hosts results in selection of a myriad of conformations, some of which stabilize becoming distinct strains, while others are temporary. Temporary conformations appear to revert to common strains (i.e., Wisc-1).

## Conclusions and perspective

The generation of new variants as a CWD strain is transmitted through a population is not uncommon and can result in variants with novel characteristics. CWD is no exception. To date, 10 CWD strains have been identified, strains that can impact incubation period, distribution of the agent in the brain, biochemistry of PrP^CWD^, shedding of infectivity (and environmental contamination), and, most importantly, the species barrier. A few strains currently predominate the CWD landscape but experimental data suggests these strains can evolve (Duque Velasquez et al. 2015, 2020; Hannaoui et al. 2021; Crowell et al. 2015; Telling 2022). There is little evidence the disease has spilled over into non-cervid species; however, CWD strains represent a changing dynamic, potentially affecting interspecies transmission. The diversity of strains identified, to date, and the likelihood of novel strains emerging and being selected (due to PrP^C^ polymorphisms and/or interspecies transmission) means that the biological properties of CWD need to be constantly monitored and assessed.

## References

[CR1] Agren EO, Soren K, Gavier-Widen D, Benestad SL, Tran L, Wall K, Averhed G, Doose N, Vage J, Noremark M (2021) First detection of chronic wasting disease in moose (Alces alces) in Sweden. J Wildl Dis 57:461–463 10.7589/JWD-D-20-00141 33822167

[CR2] Angers RC, Kang HE, Napier D, Browning S, Seward T, Mathiason C, Balachandran A, McKenzie D, Castilla J, Soto C, Jewell J, Graham C, Hoover EA, Telling GC (2010). Prion strain mutation determined by prion protein conformational compatibility and primary structure. Science.

[CR3] Angers RC, Seward TS, Napier D, Green M, Hoover E, Sprake T, O'Rourke K, Balachandran A, Telling GC (2009). Chronic wasting disease prions in elk antler velvet. Emerg Infect Dis.

[CR4] Arifin MI, Staskevicius A, Shim SY, Huang Y-H, Fenton H, McLoughlin PD, Mitchell G, Cullingham CI, Gilch S (2020). Large-scale prion protein genotyping in Canadian caribou populations and potential impact on chronic wasting disease susceptibility. Mol Ecol.

[CR5] Baeten LA, Powers BE, Jewell JE, Spraker TR, Miller MW (2007) A natural case of chronic wasting disease in a free-ranging moose (Alces alces shirasi). J Wildl Dis 43:309–314 10.7589/0090-3558-43.2.309 17495319

[CR6] Barrio T, Filali H, Otero A, Sheleby-Elias J, Marin B, Vidal E, Beringue V, Torres JM, Groschup M, Andreoletti O, Badiol JJ, Bolea R (2020). Mixtures of prion substrains in natural scrapie cases revealed by ovinised murine models. Sci Rep.

[CR7] Bartz JC, Bessen RA, McKenzie D, Marsh RF, Aiken JM (2000). Adaptation and selection of prion protein strain conformations following interspecies transmission of transmissible mink encephalopathy. J Virol.

[CR8] Bartz JC, Marsh RF, McKenzie DI, Aiken JM (1998). The host range of chronic wasting disease is altered on passage in ferrets. Virology.

[CR9] Bartz JC, McKenzie DI, Bessen RA, Marsh RF, Aiken JM (1994). Transmissible mink encephalopathy species barrier effect between ferret and mink: PrP gene and protein analysis. J Gen Virol.

[CR10] Baskakov IV (2014). The many shades of prion adaptation Prion.

[CR11] Benestad SL, Arsac JN, Goldmann W, Noremark M (2008) Atypical/Nor98 scrapie: properties of the agent, genetics and epidemiology. Vet Res 39:19 10.1051/vetres:2007056 18187032

[CR12] Benestad SL, Mitchell G, Simmons M, Ytrehus B, Vikoren T (2016). First case of chronic wasting disease in Europe in a Norwegian free-ranging reindeer. Vet Res.

[CR13] Benestad SL, Sarradin P, Thu B, Schonheit J, Tranulis MA, Bratberg B (2003) Cases of scrapie with unusual features in Norway and designation of a new type, Nor98. Vet Rec 153:202–208 10.1136/vr.153.7.202 12956297

[CR14] Bessen RA, Kocisko DA, Raymond GJ, Nandan S, Lansbury PT, Caughey B (1995). Non-genetic propagation of strain-specific properties of scrapie prion protein. Nature.

[CR15] Bessen RA, Marsh RF (1992). Biochemical and physical properties of the prion protein from two strains of the transmissible mink encephalopathy agent. J Virol.

[CR16] Bian J, Christiansen JR, Moreno JA, Kane SJ, Khaychuk V, Gallegos J, Kim S, Telling GC (2019) Primary structural differences at residue 226 of deer and elk PrP dictate selection of distinct CWD prion strains in gene-targeted mice. Proc Natl Acad Sci U S A 116:12478–12487 10.1073/pnas.1903947116 PMC6589652 31147460

[CR17] Bian J, Kim S, Kane SJ, Growell J, Sun JL, Christiansen J, Saijo E, Moreno JA, DiLisio J, Burnett E, Pritzkow S, Gorski D, Soto C, Kreeger TJ, Balanchandran A, Mitchell G, Miller MW, Nonno R, Vikoren T, Vage J, Madslien K, Tran L, Vuong TT, Benestad SL, Telling GC (2021). Adaptive selection of a prion strain conformer corresponding to established North American CWD during propagation of novel emergent Norwegian strains in mice expressing elk or deer prion protein. PLoS Pathog.

[CR18] Bian J, Napier D, Khaychuck V, Angers R, Graham C, Telling G (2010). Cell-based quantification of chronic wasting disease prions. J Virol.

[CR19] Block AJ, Bartz JC (2022) Prion strains: shining new light on old concepts. Cell Tissue Res. 10.1007/s00441-022-03665-2 10.1007/s00441-022-03665-2 PMC11318079 35796874

[CR20] Brown P, McShane LM, Zanusso G, Detwiler L (2006). On the question of sporadic or atypical bovine spongiform encephalopathy and Creutzfeldt-Jakob disease Emerg Inf Dis.

[CR21] Browning SR, Mason GL (2004). SewardT, Green M, Eliason GA, Mathiason C, Miller MW, Williams ES, Hoover E, Telling GC. Transmission of prions from mule deer and elk with chronic wasting disease to transgenic mice expressing cervid PrP J Virol.

[CR22] Bruce M, Dickinson AG. (1979) Biological stability of different classes of scrapie agent. In: Prusiner SB, Hadlow WJ (ed), Slow transmissible diseases of the nervous system. vol 2. Academic Press, New York pp 71–86

[CR23] Bruce ME (1993). Scrapie strain variation and mutation. Br Med Bull.

[CR24] Bruce ME, Will RG, Ironside JW, McConnell I, Drummond D, Suttie A, McCardle L, Chree A, Hope J, Birkett C, Cousens S, Fraser H, Bostock C (1997) Transmissions to mice indicate that “new variant” CJD is caused by the BSE agent. Nature 389:498–501 10.1038/39057 9333239

[CR25] Casalone C, Zanusso G, Acutis P, Ferrari S, Capucci L, Tagliavini F, Monaco S, Caramelli M (2004). Identification of a second bovine amyloidotic spongiform encephalopathy: molecular similarities with sporadic Creutzfeldt-Jakob disease Proc Natl Acad Sci USA.

[CR26] Caughey B, Raymond GJ, Bessen RA (1998) Strain-dependent differences in beta-sheet conformations of abnormal prion protein. J Biol Chem 273:32230–32235 10.1074/jbc.273.48.32230 9822701

[CR27] Collinge J. (2010) Prion strain mutation and selection. Science 328:1111–1112 10.1126/science.1190815 20508117

[CR28] Collinge J, Sidle KC, Meads J, Ironside J, Hill AF (1996) Molecular analysis of prion strain variation and the aetiology of “new variant” CJD. Nature 383:685–690 10.1038/383685a0 8878476

[CR29] Cortez LM, Nemani SK, Duque Velasquez C, Sriraman A, Wang Y, Wille H, McKenzie D, Sim VL (2021) Asymmetric-flow field-flow fractionation of prions reveals a strain-specific continuum of quaternary structures with protease resistance developing at a hydrodynamic radius of 15 nm. PLoS Pathog 17:e1009703 10.1371/journal.ppat.1009703 PMC8270404 34181702

[CR30] Crowell J, Hughson A, Caughey B, Bessen RA (2015). Host determinants of prion strain diversity independent of prion protein genotype. J Virol.

[CR31] Dickinson AG, Meikle VM (1970) Host-genotype and agent effects in scrapie incubation: change in allelic interaction with different strains of agent. Mol Gen Genet 112:73–79 10.1007/BF00266934 5165642

[CR32] Duque Velasquez C, Kim C, Haldiman T, Kim C, Herbst A, Aiken J, Safar JG, McKenzie D (2020) Chronic wasting disease (CWD) prion strains evolve via adaptive diversification of conformers in hosts expressing prion protein polymorphisms. J Biol Chem 295:4985–5001 10.1074/jbc.RA120.012546 PMC7152757 32111742

[CR33] Duque Velasquez C, Kim C, Herbst A, Daude N, Garza MC, Wille H, Aiken J, McKenzie D (2015). Deer Prion Proteins Modulate the Emergence and Adaptation of Chronic Wasting Disease Strains. J Virol.

[CR34] Falsig J, Aguzzi A (2008). The prion organotypic slice culture assay–POSCA. Nat Protoc.

[CR35] Fediaevsky A, Maurella C, Noremark M, Ingravalle F, Thorgeirsdottir S, Orge L, Poizat R, Hautaniemi M, Liam B, Calavas D, Ru G, Hopp P (2010) The prevalence of atypical scrapie in sheep from positive flocks is not higher than in the general sheep population in 11 European countries. BMC Vet Res 6:9 10.1186/1746-6148-6-9 PMC2832631 20137097

[CR36] Fraser H, Dickinson AG (1967). Distribution of experimental induced scrapie lesions in the brain. Nature.

[CR37] Gavier‐Widen D (2019) CWD data from Sweden. Message to Angel Ortiz Pelaez. EFSA J. 10.2903/j.efsa.2019.5863

[CR38] Green KM, Browning SR, Seward TS, Jewell JE, Ross DL, Green MA, Williams ES, Hoover EA, Telling GC (2008) The elk PRNP codon 132 polymorphism controls cervid and scrapie prion propagation. J Gen Virol 89:598–608 10.1099/vir.0.83168-0 18198392

[CR39] Guere ME, Vage J, Tharaldsen H, Benestad SL, Vikoren T, Madslien K, Hopp P, Rolandsen CM, Roed KH, Tranulis MA (2020) Chronic wasting disease associated with prion protein gene (PRNP) variation in Norwegian wild reindeer (Rangifer tarandus). Prion 14:1–10 10.1080/19336896.2019.1702446 PMC6959294 31852336

[CR40] Haldiman T, Kim C, Cohen Y, Chen W, Blevins J, Qing L, Cohen ML, Langeveld J, Telling GC, Kong Q, Safar JG (2013). Co-existence of distinct prion types enables conformational evolution of human PrPSc by competitive selection. J Biol Chem.

[CR41] Hannaoui S, Amidian S, Cheng YC, Duque Velasquez C, Dorosh L, Law S, Telling G, Stepanova M, McKenzie D, Wille H, Gilch S (2017). Destabilizing polymorphism in cervid prion protein hydrophobic core determines prion conformation and conversion efficiency. PLoS Pathog.

[CR42] Hannaoui S, Triscott E, Duque Velasquez C, Chang SC, Arifin MI, Zemlyankina I, Tang X, Bollinger T, Wille H, McKenzie D, Gilch S (2021) New and distinct chronic wasting disease strains associated with cervid polymorphism at codon 116 of the Prnp gene. PLoS Pathog 17:e1009795 10.1371/journal.ppat.1009795 PMC8341689 34310662

[CR43] Happ GM, Huson HJ, Beckmen KB, Kennedy LJ (2007). Prion protein genes in caribou from Alaska. J Wildl Dis.

[CR44] Heaton MP, Leymaster KA, Freking BA, Hawk DA, Smith TP, Keele JW, Snelling WM, Fox JM, Chitko-McKown CG, Laegreid WW (2003) Prion gene sequence variation within diverse groups of U.S. sheep, beef cattle, and deer. Mamm Genome 14:765–777 10.1007/s00335-003-2283-y 14722726

[CR45] Herbst A, Duque Velasquez C, Triscott E, Aiken JM, McKenzie D (2017). Chronic wasting disease strain emergence and host range expansion. Emerg Infect Dis.

[CR46] Herbst A, Wolgemuth S, Yang J, Castle AR, Moreno DM, Otero A, Aiken JM, Westaway D, McKenzie D (2022). Susceptibility of beavers to chronic wasting disease Biology.

[CR47] Hill AF, Desbruslais M, Joiner S, Sidle KC, Gowland I, Collinge J, Doey LJ, Lantos P (1997). The same prion strain causes vCJD and BSE. Nature.

[CR48] Huson HJ, Happ GM (2006) Polymorphisms of the prion protein gene (PRNP) in Alaskan moose (Alces alces gigas). Anim Genet 37:425–426 10.1111/j.1365-2052.2006.01466.x PMC1592321 16879366

[CR49] Jeffrey M, Gonzalez L (2007) Classical sheep transmissible spongiform encephalopathies: pathogenesis, pathological types and clinical disease. Neuropathol Appl Neurobiol 33:373–394 10.1111/j.1365-2990.2007.00868.x 17617870

[CR50] Johnson C, Johnson J, Clayton M, McKenzie D, Aiken J (2003). Prion protein gene heterogeneity in free-ranging white-tailed deer within the chronic wasting disease affected region of Wisconsin. J Wildl Dis.

[CR51] Johnson CJ, Herbst A, Duque-Velasquez C, Vanderloo JP, Bochsler P, Chappell R, McKenzie D (2011). Prion protein polymorphisms affect chronic wasting disease progression. PLoS One.

[CR52] Kascsak RJ, Rubenstein R, Merz PA, Carp RI, Wisniewski HM, Diringer H (1985). Biochemical differences among scrapie-associated fibrils support the biological diversity of scrapie agents. J Gen Virol.

[CR53] Khalili-Shirazi A, Summers L, Linehan J, Mallinson G, Anstee D, Hawke SH, Jackson GS, Collinge J (2005). PrP glycoforms are associated in a strain-specific ratio in native PrPSc J Gen Viol.

[CR54] Kimberlin RH (1982). Scrapie agent: prions or virinos?. Nature.

[CR55] Klohn PC, Stoltze L, Flechsig E, Enari M, Weissmann C (2003) A quantitative, highly sensitive cell-based infectivity assay for mouse scrapie prions Proc Natl Acad Sci U S A 100*:*11666–11671 10.1073/pnas.1834432100 PMC208815 14504404

[CR56] Kondru N, Manne S, Kokemuller R, Greenlee J, Greenlee MHW, Nichols T, Kong Q, Anantharam V Kanthasamy, A, Halbur P, Kanthasamy AG (2020) An Ex Vivo Brain Slice Culture Model of Chronic Wasting Disease: Implications for Disease Pathogenesis and Therapeutic. Development Sci Rep 10:7640 10.1038/s41598-020-64456-9 PMC7203233 32376941

[CR57] Kong Q, Huang S, Zou W, Vanegas D, Wang M, Wu D, Yuan J, Zheng M, Bai H, Deng H, Chen K, Jenny AL, O'Rourke K, Belay ED, Schonberger LB, Petersen RB, Sy MS, Chen SG, Gambetti P (2005). Chronic wasting disease of elk: transmissibility to humans examined by transgenic mouse models. J Neurosci.

[CR58] Korpenfelt S (2019) EFSA Scientific Opinion CWD. Message to Angel Ortiz Pelaez 18 July 2019. EFSA J. 10.2903/j.efsa.2019.5863

[CR59] Koutsoumanis K, Allende A, Alvarez-Ordonez A, Bolton D, Bover-Cid S, Chemaly M (2019). Update on chronic wasting disease (CWD) III. EFSA J.

[CR60] Kraus A, Hoyt F, Schwartz CL, Hansen B, Artikis E, Hughson AG, Raymond GJ, Race B, Baron GS, Caughey B (2021). High-resolution structure and strain comparison of infectious mammalian prions. Mol Cell.

[CR61] Kreeger TJ, Montgomery DL, Jewell JE, Schultz W, Williams ES (2006). Oral transmission of chronic wasting disease in captive Shira's moose. J Wildl Dis.

[CR62] LaFauci G, Carp RI, Meeker HC, Ye X, Kim JI, Natelli M, Cedeno M, Petersen RB, Kascsak R, Rubenstein R (2006) Passage of chronic wasting disease prion into transgenic mice expressing Rocky Mountain elk (Cervus elaphus nelsoni) PrPC J Gen Virol 87:3773–3780 10.1099/vir.0.82137-0 17098997

[CR63] Li J, Browning S, Mahal SP, Oelschlegel AM, Weissmann C. (2010) Darwinian evolution of prions in cell culture. Science 327(5967):869–872 10.1126/science.1183218 PMC2848070 20044542

[CR64] Manka, SW, Zhang W, Wenborn A, Betts J, Joiner S, Saibil HR, Collinge J, Wadsworth JDF (2022a) 2.7 Å cryo-EM structure of ex vivo RML prion fibrils. Nature Comm 13:4004 10.1038/s41467-022-30457-7 PMC9279362 35831275

[CR65] Manka, SW, Wenborn A, Betts J, Joiner S, Saibil HR, Collinge J, Wadsworth JDF (2022b) A structural basis for prion diversity bioRxiv 10.1038/s41589-022-01229-7 PMC10154210 36646960

[CR66] Meade-White K, Race B, Trifilo M, Bossers A, Gavara C, Lacasse R, Miller M, Williams E, Oldstone M, Race R, Chesebro B (2007) Resistance to chronic wasting disease in transgenic mice expressing a naturally occurring variant of deer prion protein. J Virol 81:4533–4539 10.1128/JVI.02762-06 PMC1900179 17314157

[CR67] Mitchell GB, Sigurdson CJ, O'Rourke KI, Algire J, Harrington NP, Walther I, Spraker TR, Balachandran A (2012). Experimental oral transmission of chronic wasting disease to reindeer (Rangifer tarandus tarandus. PLoS One.

[CR68] Moore J, Tatum T, Hwang S, Vrentas C, West Greenlee MH, Kong Q, Nicholson E, Greenlee J (2020) Novel strain of the chronic wasting disease agent isolated from experimentally inoculated elk with LL132 prion protein. Sci Rep 10:3148 10.1038/s41598-020-59819-1 PMC7035384 32081886

[CR69] Moore SJ, Vrentas CE, Hwang S, West Greenlee MH, Nicholson EM, Greenlee JJ (2018) Pathologic and biochemical characterization of PrP(Sc) from elk with PRNP polymorphisms at codon 132 after experimental infection with the chronic wasting disease agent. BMC Vet Res 14:80 10.1186/s12917-018-1400-9 PMC5845354 29523205

[CR70] Nonno R, di Bari MA, Pirisinu L, D’Agostino C, Vanni I, Chiappini B, Marcon S, Riccardi G, Tran L, Vikoren T, Bage J, Madslien K, Mitchell G, Telling GC, Benestad SL, Agrimi U (2020). Studies in bank voles reveal strain differences between chronic wasting disease prions from Norway and North America Proc Natl Acad Sci USA.

[CR71] O'Rourke KI, Spraker TR, Hamburg LK, Besser TE, Brayton KA, Knowles DP (2004). Polymorphisms in the prion precursor functional gene but not the pseudogene are associated with susceptibility to chronic wasting disease in white-tailed deer. J Gen Virol.

[CR72] O'Rourke KI, Spraker TR, Zhuang D, Greenlee JJ, Gidlewski TE, Hamir AN (2007). Elk with a long incubation prion disease phenotype have a unique PrPd profile. Neuroreport.

[CR73] Otero A, Duque Velasquez C, Johnson C, Herbst A, Bolea R, Badiola JJ, Aiken J, McKenzie D (2019) Prion protein polymorphisms associated with reduced CWD susceptibility limit peripheral PrP(CWD) deposition in orally infected white-tailed deer. BMC Vet Res 15:50 10.1186/s12917-019-1794-z PMC6360794 30717795

[CR74] Otero A, Duque Velasquez C, Aiken J, McKenzie D (2021). Chronic wasting disease: a cervid prion infection looming to spillover. Vet Res.

[CR75] Otero A, Duque Velasquez C, Aiken J, McKenzie D (2021b) White-tailed dder S96 prion protein does not support stable in vitro propagation of most common CWD strains. Sci Rep 11:11193 10.1038/s41598-021-90606-8 PMC8160261 34045540

[CR76] Parchi P, Castellani R, Capellari S, Ghetti B, Young K, Chen SG, Farlow M, Dickson DW, Sima AA, Trojanowski JQ, Petersen RB, Gambetti P (1996). Molecular basis of phenotypic variability in sporadic Creutzfeldt-Jakob disease. Ann Neurol.

[CR77] Parchi P, Zou W, Wang W, Brown P, Capellari S, Ghetti B, Kopp N, Schulz-Schaeffer WJ, Kretzschmar HA, Head MW, Ironside JW, Gambetti P, Chen SG (2000). Genetic influence on the structural variations of the abnormal prion protein Proc Natl Acad Sci USA.

[CR78] Pattison IH, Millson GC (1961). Scrapie produced experimentally in goats with special reference to the clinical syndrome. J Comp Pathol.

[CR79] Peden AH, Suleiman S, Barria MA. (2021) Understanding intra-species and inter-species prion conversion and zoonotic potential using protein misfolding cyclic amplification. Front Aging Neurosci 13:716452 10.3389/fnagi.2021.716452 PMC8368127 34413769

[CR80] Perrott MR, Sigurdson CJ, Mason GL, Hoover EA (2012) Evidence for distinct chronic wasting disease (CWD) strains in experimental CWD in ferrets. J Gen Virol 93:212–221 10.1099/vir.0.035006-0 PMC3352335 21918005

[CR81] Pirisinu L, Tran L, Chiappini B, Vanni I, Di Bari MA, Vaccari G, Vikoren T, Madslien KI, Vage J, Spraker T, Mitchell G, Balachandran A, Baron T, Casalone C, Rolandsen CM, Roed KH, Agrimi U, Nonno R, Benestad SL (2018). Novel Type of Chronic Wasting Disease Detected in Moose (Alces alces). Norway Emerg Infect Dis.

[CR82] Prusiner SB (1982). Novel proteinaceous infectious particles cause scrapie Science.

[CR83] Prusiner SB, Scott M, Foster D, Pan KM, Groth D, Mirenda C, Torchia M, Yang SL, Serban D, Carlson GA, Hoppe PC, Westaway D, DeArmond SJ (1990). Transgenetic studies implicate interactions between homologous PrP isoforms in scrapie prion replication. Cell.

[CR84] Raymond GJ, Bossers A, Raymond LD, O'Rourke KI, McHolland LE, Bryant PK 3rd, Miller MW, Williams ES, Smits M, Caughey B (2000) Evidence of a molecular barrier limiting susceptibility of humans, cattle and sheep to chronic wasting disease. EMBO J 19:4425–4430 10.1093/emboj/19.17.4425 PMC302048 10970836

[CR85] Raymond GJ, Raymond LD, Meade-White KD, Hughson AG, Favara C, Gardner D, Williams ES, Miller MW, Race RE, Caughey B (2007). Transmission and adaptation of chronic wasting disease to hamsters and transgenic mice: evidence for strains. J Virol.

[CR86] Robinson SJ, Samuel MD, O’Rourke KI, Johnson CJ (2012). The role of genetics in chronic wasting disease of North American cervids. Prion.

[CR87] Saborio GP, Permanne B, Soto C (2001). Sensitive detection of pathological prion protein by cyclic amplification of protein misfolding. Nature.

[CR88] Safar J, Wille H, Itri V, Groth D, Serban H, Torchia M, Cohen FE, Prusiner SB (1998) Eight prion strains have PrP(Sc) molecules with different conformations. Nat Med 4:1157–1165 10.1038/2654 9771749

[CR89] Safar JG, Scott M, Monaghan J, Deering C, Didorenko S, Vergara J, Ball H, Legname G, Leclerc E, Solforosi L, Serban H, Groth D, Burton DR, Prusiner SB, Williamson RA (2002). Measuring prions causing bovine spongiform encephalopathy or chronic wasting disease by immunoassays and transgenic mice. Nat Biotechnol.

[CR90] Safar JG, Xiao X, Kabir ME, Chen S, Kim C, Haldiman T, Cohen Y, Chen W, Cohen ML, Surewicz WK. (2015) Structural elements of phenotypic diversity and replication rates of human prions. PLoS Pathogens 11:e1004832 10.1371/journal.ppat.1004832 PMC4397081 25875953

[CR91] Schmitz M, Cramm M, Llorens F, Muller-Cramm D, Collins S, Atarashi R, Satoh K, Orru CD, Groveman BR, Zafar S, Schulz-Schaeffer WJ, Caughey B, Zerr I (2016). The real-time quaking-induced conversion assay for detection of human prion disease and study of other protein misfolding diseases. Nat Protoc.

[CR92] Scott M, Foster D, Mirenda C, Serban D, Coufal F, Walchli M, Torchia M, Groth D, Carlson G, DeArmond SJ, Westaway D, Prusiner SB (1989). Transgenic mice expressing hamster prion protein produce species-specific scrapie infectivity and amyloid plaques. Cell.

[CR93] Sigurdson CJ, Mathiason CK, Perrott MR, Eliason GA, Spraker TR, Glatzel M, Manco G, Bartz JC, Miller MW, Hoover EA (2008) Experimental chronic wasting disease (CWD) in the ferret. J Comp Pathol 138:189–196 10.1016/j.jcpa.2008.01.004 18387626

[CR94] Stockman S (1926). Contribution to the study of the disease known as scrapie J Comp Pathol Ther.

[CR95] Taraboulos A, Jendroska K, Serban D, Yang SL, DeArmond SJ, Prusiner SB (1992). Regional mapping of prion proteins in brain Proc Natl Acad Sci USA.

[CR96] Telling GC (2022). The shape of things to come: structural insights into how prion proteins encipher heritable information Nat Comm.

[CR97] Telling GC, Parchi P, DeArmond SJ, Cortelli P, Montagna P, Gabizon R, Mastrianni J, Lugaresi E, Gambetti P, Prusiner SB (1996). Evidence for the conformation of the pathologic isoform of the prion protein enciphering and propagating prion diversity. Science.

[CR98] Tranulis MA, Gavier-Widen D, Vage J, Noremark M, Korpenfelt S-L, Hautaniemi M, Pirisinu L, Nonno R, Benestad SL (2021). Chronic wasting disease in Europe: new strains on the horizon. Acta Veterinaria Scandinavica.

[CR99] van der Merwe J, Aiken J, Westaway D, McKenzie D (2015) The standard scrapie cell assay: development, utility and prospects. Viruses 7:180–198 10.3390/v7010180 PMC4306833 25602372

[CR100] Vikoren, T, Vage J, Madslien K, Roed K, Rolandsen CM, Tran L, Hopp P, Veiberg V, Heum M, Moldal T, das Neves CG, Handeland K, Ytrehus B, Kolbjornsen O, Wisloff H, Terland R, Saure B, Dessen KM, Gjerden Svendsen S, Nordvik BS, Benestad SL (2019) First detection of chronic wasting disease in a wild red deer (*Cervus elaphus*) in Europe. J Wildl Dis 55:970–972 30920905

[CR101] Wells GA, Scott AC, Johnson CT, Gunning RF, Hancock RD, Jeffrey M, Dawson M, Bradley R (1987). A novel progressive spongiform encephalopathy in cattle Vet. Record.

[CR102] Wik L, Mikko S, Klingeborn M, Steen M, Simonsson M, Linne T (2012) Polymorphisms and variants in the prion protein sequence of European moose (Alces alces), reindeer (Rangifer tarandus), roe deer (Capreolus capreolus) and fallow deer (Dama dama) in Scandinavia. Prion 6:256–260 10.4161/pri.19641 PMC3399539 22441661

[CR103] Williams ES (2005). Chronic wasting disease Vet Pathol.

[CR104] Williams ES, Miller MW (2002). Chronic wasting disease in deer and elk in North America. Rev Sci Tech.

